# Air vs. Road Decision for Endovascular Clot Retrieval in a Rural Telestroke Network

**DOI:** 10.3389/fneur.2020.00628

**Published:** 2020-07-17

**Authors:** Shyam Gangadharan, Thomas Lillicrap, Ferdinand Miteff, Pablo Garcia-Bermejo, Thomas Wellings, Billy O'Brien, James Evans, Khaled Alanati, Christopher Levi, Mark W. Parsons, Andrew Bivard, Carlos Garcia-Esperon, Neil J. Spratt

**Affiliations:** ^1^Department of Neurology, John Hunter Hospital, University of Newcastle, Newcastle, NSW, Australia; ^2^Hunter Medical Research Institute, University of Newcastle, Newcastle, NSW, Australia; ^3^Department of Neurology, Gosford Hospital, Gosford, NSW, Australia; ^4^Department of Neurology, Melbourne Brain Centre at the Royal Melbourne Hospital, University of Melbourne, Parkville, VIC, Australia

**Keywords:** telemedicine, decision, retrieval, thrombectomy, rural

## Abstract

**Background and Purpose:** Telestroke aims to increase access to endovascular clot retrieval (ECR) for rural areas. There is limited information on transfer workflow for ECR in rural settings. We sought to describe the transfer metrics for ECR in a rural telestroke network with respect to decision making.

**Methods:** A retrospective cohort study was employed on consecutive patients transferred to the comprehensive stroke center (CSC) for ECR in a rural hub-and-spoke telestroke network between April 2013 and October 2019, by road or air. Key time-based metrics were analyzed.

**Results:** Sixty-two patients were included. Mean age was 66 years [standard deviation (SD), 14] and median National Institutes of Health Stroke Scale 13 [interquartile range (IQR), 8–18]. Median rural-hospital-door-to-CSC-door (D2D) was 308 min (IQR, 254–351), of which 68% was spent at rural hospitals [door-in-door-out (DIDO); 214 min; IQR, 171–247]. DIDO was longer for air transfers than road (*P* = 0.004), primarily because of a median 87 min greater decision-to-departure time (Decision-DO, *P* < 0.001). In multiple linear regression analysis, intubation but not thrombolysis was associated with significantly longer DIDO. The distance at which the extra speed of an aircraft made up for the delays involved in booking an aircraft was 299 km from the CSC.

**Conclusions:** DIDO is longer for air retrievals compared with road. Decision-DO represents the most important component of DIDO, being longer for air transfers. Systems for rapid transportation of rural ECR candidates need optimization for best patient outcomes, with decision support seen as a potential tool to achieve this.

## Introduction

Endovascular clot retrieval (ECR) is the standard of care in the treatment of acute stroke patients with large vessel occlusion (LVO) (1). It has been shown to be major benefits in selected patients up to 24 h (2, 3), but earlier treatment leads to greater benefit (4–6). This presents logistical challenges in Australia, because patients from widely dispersed geographic regions are eligible for this treatment, but it is offered only in limited metropolitan centers (7, 8).

Telestroke is being increasingly used in Australia to help overcome geographical disparities in access to acute stroke care (9–11). Optimal implementation of ECR through telestroke requires efficient workflow from primary hospital to the comprehensive stroke center. Door-in-door-out (DIDO) time at the primary hospital has been thought to have the greatest impact on outcome for patients with LVO being transferred for ECR, among modifiable factors (12–14). A recent study in metropolitan Australia proposed that the target time for DIDO should be shortened to 45 min (15, 16).

Despite the fact that about 29% of Australia's population live in rural and remote areas, with people in very remote areas having a mortality rate almost 1.4 times as high as in major cities (17), there is a paucity of data on transfer workflow specific to rural Australia to guide further development of ECR in these areas. We aimed to describe the transfer metrics for ECR from rural hospitals to a regional comprehensive stroke center in a telestroke network in rural Australia, with respect to key points in clinical decision making.

Clinical decision making in stroke care is complex (18), with there being a correlation between decision delay in acute stroke and both pre-hospital and in-hospital delays (19–21). In addition to transfer workflow, we extended our study to look at the relationship between transfer metrics and clinical decision making so that the results might be more readily adapted to modify routine clinical practice and potentially identify areas for decision support. We sought to compare different transport modalities. Our main hypothesis was that DIDO for air transfers would be longer than for road transfers.

## Methods

### Setting

A “hub and spoke” telestroke network was developed to support delivery of reperfusion therapy (thrombolysis and ECR) as a 24/7 service to six rural (spoke) hospitals in the Hunter New England and Mid-North Coast local health districts of New South Wales, Australia. The comprehensive stroke center hub based at John Hunter Hospital (JHH) in Newcastle, Australia, covers a population of over 1.1 million people distributed over an area of 143,120 km^2^ (slightly larger than England). The average distance between the spoke sites and the hub is 227 km (range 167–423 km). All six spoke sites have advanced imaging (including brain CT angiography and CT perfusion) capability needed to select patients for reperfusion therapies, with all spoke sites capable of administering thrombolysis. The rural spoke hospitals were equipped with telehealth cameras, and the local physicians were trained in the face, arm, speech, time (FAST) scale. Only one of the spoke hospitals had neurologists or stroke physicians on staff who could guide local decisions about in-hours thrombolysis. All other decisions about reperfusion therapy in the acute phase were made by the telestroke neurologist at the hub (JHH) after reviewing the imaging and if required assessing the patient remotely through the telehealth cameras. Neurologists at Gosford District Hospital also participated in the telestroke roster, although only JHH accepted patients for ECR.

The decision to transfer for ECR was made by the telestroke neurologist on an individualized basis taking into account standard clinical, imaging, and patient factors. From November 2017, the time window for ECR was expanded to 24 h in image-selected patients following the release of pivotal ECR trial results (2, 3). When it was deemed necessary to give thrombolysis with ECR, thrombolysis was always initiated at the spoke hospital prior to transfer for ECR. Repeat imaging was not routinely performed at the hub because of the commitment to transfer patients across very large distances after the decision for transfer was made and the good baseline selection of patients using advanced imaging (22). Road transfers occurred by road ambulance directly from the spoke hospital to JHH. Air transfers by helicopter occurred from the rural airport servicing the respective spoke hospital to the helipad at JHH. Air transfers by fixed wing occurred from the rural airport servicing the respective spoke hospital to the regional airport based 26 km from JHH, after which the transfer to JHH took place by road ambulance. All connecting transfers to the rural airports from the respective spoke hospitals in order to facilitate air retrievals occurred by road ambulance. For more details of the telestroke setup, we direct the reader to our published experience (22, 23).

### Study Design and Data Collection

We employed a retrospective cohort study design, where transfer metrics were collected on consecutive telestroke patients transferred to JHH for ECR between April 2013 and October 2019. Clinical data were collected prospectively from June 2016 and retrospectively prior to that. Patients were transferred by road or air (helicopter or fixed wing), depending on availability and hospital distance.

Data collected included baseline demographics, past medical history, National Institutes of Health Stroke Scale (NIHSS), advanced imaging characteristics, acute treatment decision, and mode of retrieval.

### Outcome Measures and Analyses

Time metrics included DIDO, defined as the duration of time from arrival to departure at the rural hospital. DIDO was divided into two segments, door-in-to-decision (DI-Decision) and decision-to-door-out (Decision-DO). Decision was defined as the time that the telestroke neurologist at JHH contacted the rural hospital regarding the decision to transfer. Door-to-door (D2D) was the time from arrival at the rural hospital to arrival at the comprehensive stroke center. We also examined the total time from decision to arrival at JHH, and from arrival at the spoke site to arrival at JHH, correcting for the distance between spoke and hub.

### Statistical Analysis

All statistical analyses were conducted in Stata Version 14.0 (StataCorp, USA). Univariate and multivariate linear regressions were performed to estimate the effect of each variable in the form of a coefficient with respect to DIDO, DI-Decision, and Decision-DO times. Adjusting each model for individual spoke site could not be done, as each spoke site had a small number of patients and almost exclusively used either air transport or road transport, making these effects difficult to separate reliably in this data set. Further, we needed to separate delays associated with air transfers from delays related to sites with reduced experience. Thus, spoke sites were grouped by experience into more experienced sites (those with more than 10 transfers) and less experienced sites (those with 10 or fewer transfers). This would also serve to correct for potentially faster triage and treatment at more experienced sites.

Ethics approval was gained from the Hunter New England Human Research Ethics Committee (HNEHREC Reference No: 13/02/20/5.06 and AU201712-15).

## Results

Between April 2013 and October 2019, 1,087 patients were assessed by telestroke, of which 568 (52%) had confirmed ischemic strokes (when mimics and hemorrhages were excluded). Of all patients with confirmed strokes, 175 had LVO (31%). Of these patients with LVO, 75 (43%) were transferred with a view to receiving ECR. Twelve of these patients were transferred to other ECR centers owing to logistic factors relating to transport, weather, or unavailability of ECR at JHH. One patient was transferred to JHH who did not eventually receive ECR owing to established infarct found on repeat brain imaging performed at JHH due to severe unexpected delays during that transfer. A sample of 62 patients who underwent ECR at JHH was finally included in this analysis (Table 1). Mean age was 66 years [standard deviation (SD), 14 years], 34 (55%) were male, and median NIHSS was 13 [interquartile range (IQR), 8 to 18]. There were 42 air transfers (33 by helicopter and 9 by fixed wing) and 20 road transfers. The groups were well-matched overall.

**Table 1:** Baseline characteristics of different groups.

	**All transfers**	**Road transfers**	**Air transfers**	***P***
Number (No.) of patients	62	20	42	
Age, mean [SD], years	66 (14)	69 (11)	65 (15)	0.27
Sex				
Men, No. (%)	34 (55%)	11 (55%)	23 (55%)	0.99
Women, No. (%)	28 (45%)	9 (45%)	19 (45%)	
Medical history, No. (%)				
Hypertension	28 (45%)	10 (50%)	18 (43%)	0.60
Hypercholesterolemia	10 (16%)	1 (5%)	9 (21%)	0.10
Diabetes mellitus	8 (13%)	3 (15%)	5 (12%)	0.25
Prior stroke/TIA	8 (13%)	3 (15%)	5 (12%)	0.73
Atrial fibrillation	13 (21%)	2 (10%)	11 (26%)	0.14
Ischemic heart disease	9 (15%)	4 (20%)	5 (12%)	0.40
Smoker	10 (16%)	6 (30%)	4 (10%)	0.04
Prestroke mRS, No. (%)				
0–2	61 (98%)	20 (100%)	41 (98%)	0.49
3–4	1 (2%)	0 (0%)	1 (2%)	
NIHSS score, median [IQR]	13 (8-18)	9 (6-16)	14 (10-18)	0.25
Intubated, No. (%)	4 (6%)	0 (0%)	4 (10%)	0.15
Thrombolyzed, No. (%)	25 (40%)	5 (25%)	20 (48%)	0.09
Occlusion location, No. (%)				
ICA	4 (6%)	1 (5%)	3 (7%)	0.75
M1 MCA	37 (60%)	13 (65%)	24 (57%)	0.55
M2 MCA	7 (11%)	4 (20%)	3 (7%)	0.14
Basilar	5 (8%)	1 (5%)	4 (10%)	0.54

*ICA, internal carotid artery; IQR, interquartile range; MCA, middle cerebral artery; mRS, modified Rankin Scale; NIHSS, National Institutes of Health Stroke Scale; SD, standard deviation; TIA, transient ischemic attack*.

### Transfer Metrics

Median D2D time was 308 min (IQR, 254 to 351). Median DIDO time was 214 min (IQR, 171 to 247). On average, 68% (SD, 10%) of D2D time was spent at the spoke hospital, and only 32% (SD, 10%) in transit. Median DI-Decision time was 70 min (IQR, 54 to 92). Median Decision-DO time was 130 min (IQR, 82 to 182). Median time from ED arrival at spoke sites to first telestroke neurologist contact was 26 min (IQR, 16 to 43).

### Comparison of Air and Road Transfers

Spoke sites nearest to JHH transferred patients primarily by road, whereas more distant sites transferred patients primarily by air, with only two patients transferred by the method not normally used by their respective spoke site (Table 2).

**Table 2:** Distribution of transfers for rural spoke sites.

**Hospital**	**Distance to John Hunter Hospital (km)**	**Road transfers**	**Air transfers**	**Total transfers**
Taree	167	19	1	20
Tamworth	219	0	8	8
Armidale	267	0	12	12
Port Macquarie	198	1	10	11
Coffs Harbour	319	0	10	10
Moree	423	0	1	1
More experienced group (>10 transfers)		20	23	43
Less experienced group (≤10 transfers)		0	19	19

Air transfer was associated with a delay of 64 min in DIDO time (*P* = 0.004). This was entirely due to its association with longer Decision-DO times by 87 min (*P* < 0.001).

**Figure 1:**
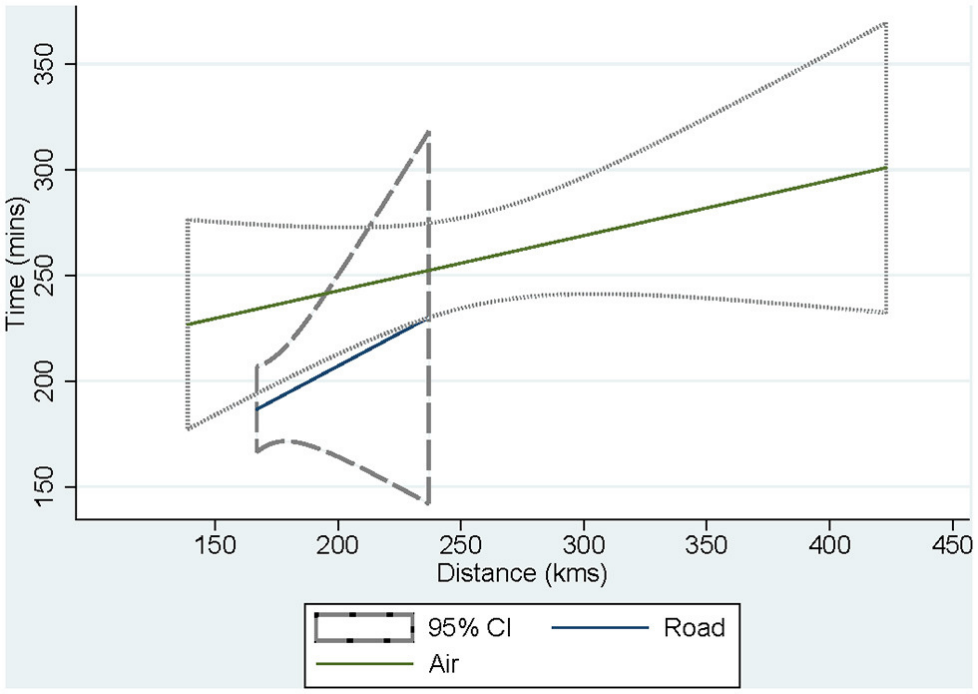
Comparison through univariate regression analysis of decision to arrival time at John Hunter Hospital for air and road transfers by distance. Extrapolating from these separate regressions suggested that the distance at which the extra speed of an aircraft made up for the delays involved in booking an aircraft was 299 km.

Separate univariate regressions of the time from decision to arrival at JHH against distance for air travel and road travel were performed and plotted (Figure 1). Extrapolating from these regressions suggested that the distance at which the extra speed of an aircraft made up for the delays involved in booking an aircraft was 299 km.

### Other Factors Which Might Influence Workflow

Differences in the level of experience between individual hospitals, that is, between the more experienced and less experienced sites (Table 2), were not significant with respect to key transfer metrics.

Intubation was associated with a delay of 242 min in DIDO and a delay of 229 min in the DI-Decision time but was not significantly associated with the Decision-DO time (*P* = 0.61, Table 3).

**Table 3:** Effects of selected variables on components of DIDO in adjusted analysis.

**Variable**	**DIDO coefficient (min) (95% CI) *P***	**DI-Decision coefficient (min) (95% CI) *P***	**Decision-DO coefficient (min) (95% CI) *P***
Experience (>10 patients transferred)	8.2 (−39.1 to 55.6) *P* = 0.73	0.9 (−29.7 to 31.5) *P* = 0.95	17.0 (−16.1 to 50.2) *P* = 0.31
Air transport	63.5 (20.8 to 106.2) *P* = 0.004	−15.7 (−43.3 to 12.0) *P* = 0.26	87.4 (56.3 to 118.5) *P* < 0.001
Intubated Note *N* = 4, all transported by air	242.3 (165.0 to 319.7) *P* < 0.001	229.2 (179.2 to 279.3) *P* < 0.001	14.6 (−42.1 to 71.2) *P* = 0.61
Thrombolyzed	11.0 (−26.2 to 48.2) *P* = 0.56	−26.3 (−50.4 to −2.3) *P* = 0.033	33.8 (7.1 to 60.5) *P* = 0.014
Adjusted *R*^2^	0.5134	0.6523	0.4313
*P* overall model	<0.001	<0.001	<0.001

*DIDO, door-in-door-out; DI-Decision, door-in-to-decision; Decision-DO, decision-to-door-out*.

Thrombolysis was associated with faster DI-Decision times by 26 min but slower Decision-DO times by 34 min, which canceled each other out with respect to DIDO times (meaning thrombolysis was not significantly associated with DIDO, *P* = 0.56).

## Discussion

We found that air transfer was associated with a significant delay in DIDO time as compared with road. The main reason for this appeared to be the longer Decision-DO with air travel, which was probably attributable to multiple logistical factors that increased the complexity of organizing air retrieval. Our study indicates that Decision-DO appears to be a key component of DIDO, which has special relevance to the rural telestroke setup. While training and systems are already being used to minimize the DI-Decision time, which was also found to be too long in our study, Decision-DO in particular represents an important target for future optimization of transfer workflow for ECR in our area.

Our analysis suggests that the critical distance at which the speed of air travel makes up for the delays in booking aircraft is ~300 km in our telestroke network, although there is considerable uncertainty about the exact figure, owing to a lack of overlap in the distances covered by air transfers compared with road transfers. Nevertheless, this figure may serve in future as a guide to base decisions about allocation of retrieval resources. For instance, when applied to other health service networks with comparable demographics and infrastructure to ours, transfers below a similar threshold may be prioritized for road rather than air retrieval (24–27).

We discovered that DIDO in our study was 214 min, more than double that published in other metropolitan Australian networks (15, 16). Potential reasons for our relatively long DIDO time include the much larger distances needed to be traversed by retrieval services in our network (hence affecting transport arrival times to rural spoke sites) as well as the resource and workforce limitations inherent in the rural setting.

Intubation was associated with a significant delay in DIDO, whereas thrombolysis was not. Our results are in line with previous studies in which thrombolysis did not have a detrimental impact on workflow of ECR transfers (14–16). However, although thrombolysis had no clear effect on DIDO in our study, it was associated with longer Decision-DO, which was compensated by shorter DI-Decision. This indicates that patients who are identified as potentially suitable for thrombolysis are triaged and treated quickly in our rural spoke sites relative to those who present later. The fact that the Decision-DO time is extended suggests that the process of thrombolysis may actually delay transfers slightly. This effect might be potentially reduced by using tenecteplase instead of alteplase for thrombolysis, which is just a bolus dose and not an infusion.

Although there may be too many variables in the telestroke-guided retrieval process to fully control for, our study raises a few potential roles for decision support in this process, with the overarching goal of reducing delays. One such role might be to integrate the entire retrieval process, so there is a uniform approach for all transfers. For example, once the decision to transfer for ECR has been made in a particular case, a decision support system may work via a platform that helps to coordinate the retrieval process in real time and generate the most appropriate transfer strategy (taking into account factors such as distance, current weather conditions, availability of aircraft and landing stations, and need for intubation before transfer).

Another role for decision support might be in helping to activate the retrieval service. Ideally, the retrieval system could be activated for suspected LVO patients even before the formal decision by the telestroke neurologist. For example, decision support could facilitate ED and ambulance staff to activate the retrieval pathway even prior to consultation with the telestroke neurologist if certain clinical or imaging criteria are met, with the capacity to abort the transfer later if needed. This may serve to reduce Decision-DO in our population, although its implementation is likely to be hindered by transport limitations.

Besides the limited generalizability of our results due to small sample size and heterogeneity of our rural spoke sites, it was beyond the scope of our analysis to measure patient outcomes. Although we did not specifically look at patient outcomes, we instead studied key time metrics associated with DIDO, and by inference, the optimization of these metrics would likely serve to improve patient outcomes. The basis for this is that it is already well-established that delays in DIDO equate to delayed recanalization and hence are detrimental to patient outcomes (12). For similar reasons, we could not adequately compare helicopter and fixed wing retrievals. Some of our data were collected retrospectively. We were unable to account for less predictable factors, which could potentially affect transfers such as the effects of peak-hour traffic, aircraft availability, and refueling protocols.

In conclusion, DIDO in rural areas is longer than described in metropolitan areas. DIDO is longer for air retrievals compared with road. Decision-DO time represents the most important component of DIDO and hence a potential target for future interventions to improve transfer workflow. Decision-DO varies depending upon transport modality, being longer for air travel than road. Systems for rapid transportation of rural ECR candidates need optimization to minimize delays to treatment and ensure best patient outcomes, with decision support seen as a potential tool to achieve this.

## Data Availability Statement

The datasets generated for this study will not be made publicly available. The full data-set contains potentially identifiable patient data. Request to access the data can be directed to the corresponding author.

## Ethics Statement

The studies involving human participants were reviewed and approved by Hunter New England Human Research Ethics Committee. Written informed consent for participation was not required for this study in accordance with the national legislation and the institutional requirements.

## Author Contributions

SG: took the lead in writing the manuscript, in consultation with NS, CG-E, and TL. NS and CG-E: made an equal contribution to the article as joint senior authors and conceived the framework for the project with input from CL and together oversaw the overall operation of the telestroke network. In addition, TL, FM, PG-B, TW, BO'B, JE, KA, CG-E, and NS: collected and collated data for the article. TL conducted the statistical analyses. MP and AB: oversaw the imaging protocols. All authors contributed to designing and writing and the conception of the manuscript on behalf of the Northern NSW Telestroke investigators.

## Members (Besides Authors) of Northern NSW Telestroke Investigators

Ms Rachel Peake, Dr James Hughes, Dr Lisa Dark, Dr Nick Ryan, Dr Matt Shepherd, Dr Osama Ali, Dr Hugh Reid, Ms Fiona Minett, Ms Jaclyn Birnie, Ms Amanda Buzio, Dr Iain Bruce, Dr Alan Tankel, Ms Kim Parrey, Dr Matthew Kinchington, Dr Elizabeth Pepper, Dr Andre Loiselle, Dr James Thomas, Dr Jessica Stabler, Dr Mohammad Amin, Ms Michelle Russell, Ms Angela Royan, Mr Brett Roworth, Dr Mary Morgan.s.

## Conflict of Interest

The authors declare that the research was conducted in the absence of any commercial or financial relationships that could be construed as a potential conflict of interest.
